# Supplementary material for: Hot spot prediction in protein-protein interactions by an ensemble system
Source: BMC Syst Biol. 2018 Dec 31;12(Suppl 9):132. doi: 10.1186/s12918-018-0665-8 (PMC6311905; doi:10.1186/s12918-018-0665-8)
Supplement: Supplementary file 2 — All relevant MATLAB code. A simple Matlab implement of our predictor. (ZIP 19,742 kb) [file 12918_2018_665_MOESM2_ESM.zip › Additional file 2/Read ASA/ASA website/dbMPIKT/1N8ZB.html]

 NetSurfP - Protein Surface Accessibility and Secondary Structure Predictions

  

|  |  |  |
| --- | --- | --- |
|  |  | NetSurfP - Protein Surface Accessibility and Secondary Structure Predictions  Technical University of Denmark |

  


---

```
# For publication of results, please cite:
# A generic method for assignment of reliability scores applied to solvent accessibility predictions.
# Bent Petersen, Thomas Nordahl Petersen, Pernille Andersen, Morten Nielsen and Claus Lundegaard
# BMC Structural Biology 2009, 9:51 doi:10.1186/1472-6807-9-51
#
# Column 1: Class assignment - B for buried or E for Exposed - Threshold: 25% exposure, but not based on RSA
# Column 2: Amino acid
# Column 3: Sequence name
# Column 4: Amino acid number
# Column 5: Relative Surface Accessibility - RSA
# Column 6: Absolute Surface Accessibility
# Column 7: Z-fit score for RSA prediction
# Column 8: Probability for Alpha-Helix
# Column 9: Probability for Beta-strand
# Column 10: Probability for Coil
E E  1N8ZB                  1    0.823 143.813   1.345   0.003   0.003   0.994
B V  1N8ZB                  2    0.205  31.570  -0.234   0.004   0.514   0.481
E Q  1N8ZB                  3    0.430  76.816   1.282   0.002   0.816   0.182
B L  1N8ZB                  4    0.154  28.216   0.676   0.002   0.816   0.182
E V  1N8ZB                  5    0.384  58.975   0.535   0.021   0.756   0.223
B E  1N8ZB                  6    0.243  42.382   0.807   0.004   0.514   0.481
E S  1N8ZB                  7    0.418  49.025  -0.469   0.020   0.205   0.775
E G  1N8ZB                  8    0.501  39.468  -1.853   0.018   0.088   0.893
E G  1N8ZB                  9    0.525  41.286  -2.269   0.018   0.047   0.935
E G  1N8ZB                 10    0.516  40.617  -1.734   0.020   0.205   0.775
E L  1N8ZB                 11    0.399  73.075  -0.785   0.022   0.359   0.619
E V  1N8ZB                 12    0.303  46.510   0.697   0.021   0.451   0.528
E Q  1N8ZB                 13    0.426  76.012   1.218   0.021   0.279   0.699
E P  1N8ZB                 14    0.459  65.061  -0.513   0.018   0.047   0.935
E G  1N8ZB                 15    0.610  47.991  -1.979   0.005   0.045   0.951
E G  1N8ZB                 16    0.412  32.417  -0.626   0.004   0.138   0.858
E S  1N8ZB                 17    0.430  50.373  -0.422   0.004   0.420   0.576
B L  1N8ZB                 18    0.151  27.611   0.776   0.002   0.816   0.182
E R  1N8ZB                 19    0.411  94.211   0.857   0.001   0.900   0.099
B L  1N8ZB                 20    0.074  13.641   0.373   0.001   0.900   0.099
B S  1N8ZB                 21    0.168  19.631   0.617   0.001   0.900   0.099
B C  1N8ZB                 22    0.038   5.293  -0.151   0.001   0.900   0.099
B A  1N8ZB                 23    0.287  31.605   0.669   0.001   0.900   0.099
B A  1N8ZB                 24    0.095  10.436  -0.395   0.002   0.816   0.182
B S  1N8ZB                 25    0.177  20.791  -0.575   0.004   0.514   0.481
B G  1N8ZB                 26    0.231  18.180  -1.942   0.004   0.197   0.799
B F  1N8ZB                 27    0.242  48.610  -0.811   0.005   0.336   0.660
B N  1N8ZB                 28    0.185  27.040  -0.718   0.004   0.514   0.481
B I  1N8ZB                 29    0.110  20.387  -0.893   0.021   0.451   0.528
B K  1N8ZB                 30    0.211  43.485  -0.952   0.021   0.279   0.699
B D  1N8ZB                 31    0.300  43.288  -1.146   0.018   0.088   0.893
B T  1N8ZB                 32    0.119  16.547  -0.099   0.019   0.141   0.840
B Y  1N8ZB                 33    0.086  18.485   0.215   0.022   0.359   0.619
B I  1N8ZB                 34    0.030   5.531   0.160   0.023   0.655   0.322
B H  1N8ZB                 35    0.054   9.914   0.061   0.021   0.756   0.223
B W  1N8ZB                 36    0.038   9.067   0.285   0.021   0.756   0.223
B V  1N8ZB                 37    0.039   5.979   0.147   0.021   0.756   0.223
B R  1N8ZB                 38    0.130  29.770   0.284   0.023   0.655   0.322
B Q  1N8ZB                 39    0.097  17.271   0.342   0.022   0.359   0.619
B A  1N8ZB                 40    0.204  22.437   0.856   0.005   0.045   0.951
E P  1N8ZB                 41    0.361  51.226   0.717   0.005   0.015   0.979
E G  1N8ZB                 42    0.582  45.780  -1.650   0.018   0.019   0.964
E K  1N8ZB                 43    0.399  82.054   0.659   0.018   0.019   0.964
E G  1N8ZB                 44    0.440  34.620  -0.460   0.018   0.088   0.893
B L  1N8ZB                 45    0.157  28.692   1.071   0.021   0.279   0.699
B E  1N8ZB                 46    0.090  15.671  -0.437   0.004   0.616   0.381
B W  1N8ZB                 47    0.046  11.183   0.125   0.001   0.900   0.099
B V  1N8ZB                 48    0.023   3.597   0.950   0.001   0.959   0.040
B A  1N8ZB                 49    0.017   1.884  -0.092   0.001   0.959   0.040
B R  1N8ZB                 50    0.076  17.404  -0.573   0.001   0.959   0.040
B I  1N8ZB                 51    0.022   4.089   0.867   0.001   0.959   0.040
B Y  1N8ZB                 52    0.151  32.247   0.517   0.002   0.816   0.182
B P  1N8ZB                 53    0.261  37.078  -1.253   0.005   0.336   0.660
E T  1N8ZB                 54    0.529  73.400  -1.800   0.005   0.015   0.979
E N  1N8ZB                 55    0.608  88.938  -2.816   0.003   0.003   0.994
E G  1N8ZB                 56    0.556  43.734  -2.371   0.018   0.019   0.964
E Y  1N8ZB                 57    0.481 102.683  -0.609   0.018   0.047   0.935
E T  1N8ZB                 58    0.319  44.301  -0.136   0.020   0.205   0.775
E R  1N8ZB                 59    0.424  96.982   0.066   0.021   0.279   0.699
B Y  1N8ZB                 60    0.192  41.137   0.504   0.020   0.205   0.775
B A  1N8ZB                 61    0.212  23.362  -0.237   0.018   0.088   0.893
E D  1N8ZB                 62    0.644  92.786  -0.638   0.018   0.047   0.935
E S  1N8ZB                 63    0.417  48.908  -0.140   0.018   0.047   0.935
B V  1N8ZB                 64    0.091  13.941  -0.155   0.019   0.141   0.840
E K  1N8ZB                 65    0.401  82.486   0.736   0.004   0.138   0.858
B G  1N8ZB                 66    0.230  18.125  -1.692   0.005   0.336   0.660
B R  1N8ZB                 67    0.215  49.304   0.627   0.003   0.718   0.279
B F  1N8ZB                 68    0.056  11.199  -0.399   0.001   0.900   0.099
B T  1N8ZB                 69    0.109  15.077   0.618   0.001   0.959   0.040
B I  1N8ZB                 70    0.043   7.900   0.693   0.001   0.959   0.040
B S  1N8ZB                 71    0.088  10.349   0.629   0.001   0.900   0.099
B A  1N8ZB                 72    0.229  25.269   0.635   0.004   0.616   0.381
B D  1N8ZB                 73    0.207  29.858  -0.318   0.004   0.514   0.481
E T  1N8ZB                 74    0.419  58.060  -0.136   0.018   0.088   0.893
B S  1N8ZB                 75    0.246  28.831  -1.843   0.018   0.047   0.935
E K  1N8ZB                 76    0.510 104.989   0.547   0.018   0.088   0.893
B N  1N8ZB                 77    0.250  36.629  -0.489   0.022   0.359   0.619
B T  1N8ZB                 78    0.216  30.015   1.098   0.018   0.846   0.136
B A  1N8ZB                 79    0.065   7.130  -0.454   0.011   0.918   0.071
B Y  1N8ZB                 80    0.163  34.812   0.571   0.006   0.962   0.032
B L  1N8ZB                 81    0.046   8.404   0.635   0.011   0.918   0.071
B Q  1N8ZB                 82    0.165  29.398  -0.384   0.011   0.918   0.071
B M  1N8ZB                 83    0.067  13.387  -0.620   0.023   0.655   0.322
E N  1N8ZB                 84    0.378  55.325  -0.016   0.022   0.359   0.619
E S  1N8ZB                 85    0.397  46.517  -0.714   0.020   0.205   0.775
B L  1N8ZB                 86    0.131  24.004  -0.315   0.018   0.088   0.893
E R  1N8ZB                 87    0.489 112.027   0.960   0.018   0.088   0.893
E A  1N8ZB                 88    0.388  42.747  -0.527   0.052   0.084   0.864
E E  1N8ZB                 89    0.442  77.217  -0.754   0.053   0.043   0.903
B D  1N8ZB                 90    0.225  32.365  -0.703   0.052   0.084   0.864
B T  1N8ZB                 91    0.105  14.619   0.147   0.066   0.296   0.638
B A  1N8ZB                 92    0.037   4.088  -0.304   0.021   0.756   0.223
B V  1N8ZB                 93    0.062   9.576   0.916   0.018   0.846   0.136
B Y  1N8ZB                 94    0.037   7.864   0.643   0.018   0.846   0.136
B Y  1N8ZB                 95    0.048  10.279   0.773   0.021   0.756   0.223
B C  1N8ZB                 96    0.016   2.246   1.454   0.021   0.756   0.223
B S  1N8ZB                 97    0.029   3.422  -0.092   0.023   0.655   0.322
B R  1N8ZB                 98    0.121  27.778  -0.263   0.021   0.451   0.528
B W  1N8ZB                 99    0.216  51.948  -0.367   0.021   0.279   0.699
E G  1N8ZB                100    0.314  24.704  -1.282   0.019   0.141   0.840
E G  1N8ZB                101    0.379  29.859  -2.385   0.018   0.047   0.935
E D  1N8ZB                102    0.386  55.623  -1.723   0.018   0.047   0.935
E G  1N8ZB                103    0.377  29.646  -1.951   0.018   0.047   0.935
E F  1N8ZB                104    0.317  63.622  -1.562   0.018   0.088   0.893
B Y  1N8ZB                105    0.223  47.719  -0.577   0.018   0.088   0.893
B A  1N8ZB                106    0.115  12.662  -0.985   0.020   0.205   0.775
B M  1N8ZB                107    0.056  11.266   0.135   0.020   0.205   0.775
B D  1N8ZB                108    0.104  15.015  -0.398   0.021   0.279   0.699
B Y  1N8ZB                109    0.074  15.771  -0.357   0.020   0.205   0.775
B W  1N8ZB                110    0.049  11.857  -0.752   0.019   0.141   0.840
B G  1N8ZB                111    0.047   3.715  -1.428   0.019   0.141   0.840
B Q  1N8ZB                112    0.164  29.290  -0.189   0.004   0.085   0.910
B G  1N8ZB                113    0.114   9.003  -1.741   0.005   0.336   0.660
B T  1N8ZB                114    0.069   9.501   0.193   0.004   0.616   0.381
B L  1N8ZB                115    0.203  37.078   0.037   0.001   0.900   0.099
B V  1N8ZB                116    0.035   5.426   0.235   0.001   0.959   0.040
B T  1N8ZB                117    0.082  11.415   0.370   0.001   0.900   0.099
B V  1N8ZB                118    0.067  10.236   0.214   0.002   0.816   0.182
B S  1N8ZB                119    0.136  15.881  -0.583   0.004   0.616   0.381
B S  1N8ZB                120    0.248  29.112  -0.931   0.005   0.262   0.733
E A  1N8ZB                121    0.454  49.998  -1.663   0.018   0.088   0.893
E S  1N8ZB                122    0.450  52.752  -0.373   0.018   0.088   0.893
E T  1N8ZB                123    0.464  64.371  -1.487   0.018   0.047   0.935
E K  1N8ZB                124    0.603 124.037  -1.548   0.005   0.015   0.979
E G  1N8ZB                125    0.510  40.161  -1.720   0.005   0.015   0.979
E P  1N8ZB                126    0.402  57.001  -1.846   0.005   0.045   0.951
E S  1N8ZB                127    0.521  61.049  -1.248   0.019   0.141   0.840
E V  1N8ZB                128    0.298  45.772  -1.492   0.021   0.279   0.699
B F  1N8ZB                129    0.164  33.015   0.520   0.021   0.279   0.699
E P  1N8ZB                130    0.344  48.785  -0.741   0.005   0.262   0.733
B L  1N8ZB                131    0.169  31.017  -1.326   0.005   0.262   0.733
E A  1N8ZB                132    0.358  39.452  -1.002   0.004   0.197   0.799
E P  1N8ZB                133    0.322  45.649  -1.985   0.004   0.138   0.858
B S  1N8ZB                134    0.216  25.292  -2.321   0.004   0.085   0.910
E S  1N8ZB                135    0.458  53.666  -2.509   0.005   0.045   0.951
E K  1N8ZB                136    0.697 143.373  -2.027   0.005   0.015   0.979
E S  1N8ZB                137    0.448  52.459  -2.000   0.005   0.015   0.979
E T  1N8ZB                138    0.521  72.304  -1.159   0.003   0.003   0.994
E S  1N8ZB                139    0.565  66.183  -2.013   0.003   0.003   0.994
E G  1N8ZB                140    0.683  53.784  -1.569   0.003   0.003   0.994
E G  1N8ZB                141    0.466  36.682  -1.284   0.005   0.045   0.951
E T  1N8ZB                142    0.431  59.766   0.773   0.004   0.420   0.576
B A  1N8ZB                143    0.057   6.226   0.349   0.002   0.816   0.182
B A  1N8ZB                144    0.232  25.588   0.886   0.001   0.900   0.099
B L  1N8ZB                145    0.045   8.331  -0.420   0.001   0.959   0.040
B G  1N8ZB                146    0.054   4.242   0.043   0.001   0.900   0.099
B C  1N8ZB                147    0.029   4.114   0.156   0.002   0.816   0.182
B L  1N8ZB                148    0.157  28.838   0.469   0.001   0.900   0.099
B V  1N8ZB                149    0.068  10.482  -0.551   0.003   0.718   0.279
E K  1N8ZB                150    0.295  60.661  -0.018   0.004   0.514   0.481
E D  1N8ZB                151    0.379  54.657  -2.040   0.005   0.262   0.733
B Y  1N8ZB                152    0.228  48.745  -0.639   0.005   0.262   0.733
E F  1N8ZB                153    0.344  69.081  -0.296   0.004   0.138   0.858
B P  1N8ZB                154    0.212  30.111  -1.340   0.004   0.138   0.858
E E  1N8ZB                155    0.626 109.450  -0.160   0.004   0.138   0.858
E P  1N8ZB                156    0.436  61.854  -1.132   0.004   0.197   0.799
B V  1N8ZB                157    0.185  28.388   0.485   0.004   0.616   0.381
E T  1N8ZB                158    0.390  54.148  -0.124   0.001   0.900   0.099
B V  1N8ZB                159    0.071  10.851   0.279   0.001   0.900   0.099
B S  1N8ZB                160    0.281  32.921   0.276   0.001   0.900   0.099
B W  1N8ZB                161    0.076  18.254  -0.548   0.011   0.918   0.071
E N  1N8ZB                162    0.374  54.739   0.324   0.003   0.718   0.279
E S  1N8ZB                163    0.469  54.932  -0.741   0.004   0.420   0.576
E G  1N8ZB                164    0.516  40.641  -1.051   0.018   0.088   0.893
E A  1N8ZB                165    0.557  61.392  -0.185   0.018   0.019   0.964
E L  1N8ZB                166    0.502  91.916  -0.744   0.018   0.047   0.935
E T  1N8ZB                167    0.572  79.309  -2.232   0.018   0.047   0.935
E S  1N8ZB                168    0.591  69.300  -0.417   0.018   0.047   0.935
E G  1N8ZB                169    0.435  34.227  -0.323   0.019   0.141   0.840
B V  1N8ZB                170    0.194  29.802  -0.312   0.004   0.420   0.576
E H  1N8ZB                171    0.329  59.772   0.492   0.002   0.816   0.182
B T  1N8ZB                172    0.262  36.326  -0.218   0.018   0.846   0.136
B F  1N8ZB                173    0.128  25.649   1.378   0.021   0.756   0.223
B P  1N8ZB                174    0.160  22.690  -0.292   0.022   0.552   0.426
B A  1N8ZB                175    0.171  18.855  -1.196   0.023   0.655   0.322
B V  1N8ZB                176    0.141  21.672  -1.411   0.018   0.846   0.136
B L  1N8ZB                177    0.262  48.064  -0.165   0.003   0.718   0.279
E Q  1N8ZB                178    0.422  75.441  -0.140   0.004   0.420   0.576
E S  1N8ZB                179    0.635  74.387  -1.966   0.004   0.138   0.858
E S  1N8ZB                180    0.693  81.184  -1.596   0.005   0.045   0.951
E G  1N8ZB                181    0.420  33.015  -1.661   0.004   0.138   0.858
E L  1N8ZB                182    0.360  65.824   0.534   0.004   0.616   0.381
B Y  1N8ZB                183    0.157  33.615   0.025   0.001   0.900   0.099
B S  1N8ZB                184    0.147  17.217  -0.507   0.001   0.900   0.099
B L  1N8ZB                185    0.108  19.720  -0.623   0.001   0.959   0.040
B S  1N8ZB                186    0.191  22.373  -0.467   0.001   0.959   0.040
B S  1N8ZB                187    0.052   6.153  -0.986   0.001   0.959   0.040
B V  1N8ZB                188    0.217  33.291   0.428   0.001   0.959   0.040
B V  1N8ZB                189    0.050   7.654   0.075   0.001   0.959   0.040
E T  1N8ZB                190    0.284  39.363   0.980   0.001   0.900   0.099
B V  1N8ZB                191    0.068  10.436   1.068   0.001   0.900   0.099
E P  1N8ZB                192    0.367  52.091   1.044   0.004   0.616   0.381
E S  1N8ZB                193    0.358  41.922  -0.206   0.022   0.359   0.619
E S  1N8ZB                194    0.589  69.019  -0.873   0.021   0.451   0.528
E S  1N8ZB                195    0.634  74.258   0.404   0.005   0.262   0.733
B L  1N8ZB                196    0.192  35.210  -0.694   0.004   0.138   0.858
E G  1N8ZB                197    0.733  57.679   0.622   0.005   0.015   0.979
E T  1N8ZB                198    0.725 100.613   1.006   0.005   0.015   0.979
E Q  1N8ZB                199    0.562 100.337   1.134   0.005   0.336   0.660
E T  1N8ZB                200    0.487  67.602   1.310   0.001   0.900   0.099
B Y  1N8ZB                201    0.071  15.258   0.651   0.001   0.959   0.040
E I  1N8ZB                202    0.357  66.137   1.856   0.001   0.959   0.040
B C  1N8ZB                203    0.026   3.650   0.077   0.001   0.959   0.040
E N  1N8ZB                204    0.396  57.989   1.256   0.001   0.900   0.099
B V  1N8ZB                205    0.081  12.434  -0.417   0.001   0.900   0.099
E N  1N8ZB                206    0.568  83.214   0.678   0.001   0.900   0.099
B H  1N8ZB                207    0.191  34.707   0.236   0.004   0.514   0.481
E K  1N8ZB                208    0.619 127.431   0.321   0.005   0.045   0.951
E P  1N8ZB                209    0.728 103.374  -1.058   0.005   0.015   0.979
E S  1N8ZB                210    0.530  62.163  -0.315   0.005   0.045   0.951
E N  1N8ZB                211    0.590  86.347   0.147   0.004   0.085   0.910
E T  1N8ZB                212    0.528  73.303   0.712   0.005   0.262   0.733
E K  1N8ZB                213    0.560 115.130   1.422   0.004   0.514   0.481
B V  1N8ZB                214    0.120  18.413   0.164   0.002   0.816   0.182
E D  1N8ZB                215    0.707 101.821   0.574   0.001   0.900   0.099
E K  1N8ZB                216    0.320  65.865   1.292   0.002   0.816   0.182
E K  1N8ZB                217    0.635 130.681   0.843   0.003   0.718   0.279
E V  1N8ZB                218    0.254  39.040   0.621   0.005   0.262   0.733
E E  1N8ZB                219    0.598 104.453   0.540   0.005   0.015   0.979
E P  1N8ZB                220    0.784 111.250  -1.336   0.000   0.000   0.999

Explain the output.  Go back.
```

---
